# Childhood Cancer Survival in Brunei Darussalam

**DOI:** 10.31557/APJCP.2020.21.11.3259

**Published:** 2020-11

**Authors:** Elvynna Leong, Sok King Ong, Fadzilah Jali, Noraslinah Ramlee

**Affiliations:** 1 *Faculty of Science, Universiti Brunei Darussalam, Jln Tungku Link, Brunei Darussalam. *; 2 *Institute of Applied Data Analytics, Universiti Brunei Darussalam, Jln Tungku Link, Brunei Darussalam. *; 3 *NCD Prevention Unit, Ministry of Health, Commonwealth Drive, Brunei Darussalam. *; 4 *Early Detection & Cancer Prevention Services, Pantai Jerudong Specialist Centre, Brunei Darussalam. *

**Keywords:** Childhood cancer, survival, hazard, prognostic factor, population-based, cancer registry, Cox PH, Brunei

## Abstract

**Background:**

This study aims to determine the survival rates for children and adolescents aged 0-19 years diagnosed with childhood cancer and to evaluate the associated factors for childhood cancer survival in Brunei Darussalam.

**Methods:**

The analysis was based on de-identified data of 263 childhood cancer for the period 2002 to 2017 retrieved from a population-based cancer registry. Overall survival was estimated using the Kaplan-Meier method. Univariate analysis, using the log-rank test, was used to examine the differences in survival between groups. Multivariate analysis, using the Cox Proportional Hazard (PH) regression model, was used to estimate the hazard ratios (HRs) and select the significant associated factors for childhood cancer patients’ survival.

**Results:**

The overall 1-, 5- and 10-year survival rates for all childhood cancers combined were 79.4%, 70.0% and 68.8% respectively. The most common types of cancer were leukemias, malignant epithelial neoplasms, lymphomas and tumours of the central nervous system (CNS). The 5-year survival estimates were highest for malignant epithelial neoplasms (84.2%) while the lowest was tumours of the CNS (44.1%). Log rank tests showed significant differences in childhood cancer patients’ survival between tumour types and period of diagnosis. In the Cox PH analysis, the presence of lymphomas, gonodal and germ cell neoplasms, and malignant epithelial neoplasms compared to leukemia; children aged 1-4 and 5-9 years compared to adolescents aged 15-19 years; and periods of diagnosis in 2002-2006 and 2007-2011 compared to 2012-2017 were significantly associated with lower hazard of death in this study.

**Conclusion:**

This study provides a baseline measurement of childhood cancer survival for monitoring and evaluation of cancer control programmes, to allow planning of cancer control program strategies such as surveillance, screening, and treatment to improve childhood survival rates in Brunei Darussalam.

## Introduction

Cancer in children and adolescents aged 0-19 years was the sixth leading cause of total cancer burden globally and the ninth leading cause of childhood disease burden globally (Force et al., 2019). Although childhood cancer is rare compared to adult cancers, it has a tremendous impact on the health of children and their families, and is an important concern for public health, medical care, psychology and society (Steliarova-Foucher et al., 2017). Childhood cancer can also lead to a high burden of serious and chronic disability caused by cancer treatments (Mitra et al., 2017). It is estimated that more than 60% of childhood cancer survivors suffer from at least one chronic condition and almost 30% have severe or life-threatening conditions (Oeffinger et al., 2006). 

More than 80% of children with cancer in high-income countries survive 5-years after diagnosis, but in many low- and middle-income countries, survival rates remain significantly lower due to the need for specialized care centers, shortages of essential chemotherapy, treatment abandonment, late diagnosis and financial burden (Gupta et al., 2015). In countries with high economic status, children are more likely to have access to high quality treatment and supportive care, health insurance, receive a timely diagnosis and parents are more likely to have high level of knowledge and adherence to therapy, all of which contribute to improved childhood cancer survival rates (Park et al., 2016). In 2018, the World Health Organization (WHO) established the Global Initiative for Childhood Cancer, which aims to achieve a global survival rate of at least 60% for all children diagnosed with cancer by 2030 (World Health Organization, 2018). Increasing survival will require considerable planning by policy makers to ensure adequate resource allocation and health system function. 

The leading cause of deaths in Brunei Darussalam is from cancer, which accounts for about 19% of the total mortalities in the country (DPP, 2019). In 2019, children and adolescents made up 25.7% of the total population in Brunei Darussalam. Brunei Darussalam is a country in South East Asia with an estimated population of 459,500 where the major ethnic groups are Malay (65.8%), Chinese (10.2%) and other ethnicities (24.0%) (DEPS, 2019).

This paper aims to determine the survival rates for children and adolescents aged 0-19 years diagnosed with childhood cancer, and evaluate the associated factors for childhood cancer survival in Brunei Darussalam, enabling for comparability with population-based estimates from other countries. 

## Materials and Methods

This study was conducted using de-identified data from the Brunei Darussalam Cancer Registry (BDCR) recorded from 1st January 2002 to 31st December 2017. Only childhood cancer patients who are local citizen and permanent residents of Brunei Darussalam registered with the local health services were included in this study. Ethical approval for this study was obtained from PAPRSB Institute of Health Science Research and Ethics Committee, the Medical and Health Research Ethics committee of Ministry of Health, Brunei Darussalam [Ref: UBD/PAPRSBIHSREC/2018/149] and University Research Ethics Committee, Universiti Brunei Darussalam [Ref: UBD/OAVCR/UREC/Apr2020-03].

The data extracted from the medical records included demographic characteristics such as age at diagnosis (ages 0-14, 0, 1-4, 5-9, 10-14, and 15-19 years), gender (Male and Female), ethnicity (Malay, Chinese, Others), period of diagnosis (2002-2006, 2007-2011, and 2012-2017) and clinical characteristics such as tumour types (Leukemias, Lymphomas, Tumours of the central nervous system (CNS), Neuroblastoma, Retinoblastoma, Renal tumours, Hepatic tumours, Malignant bone tumours, Soft tissue sarcomas, Gonadal and germ neoplasms, Malignant epithelial neoplasms, Other and unspecified).

Cancer in children and adolescents aged 0-19 years is referred to as childhood cancer in this article. An age range of 0-19 years was also chosen in WHO and previous US and European studies of childhood cancer incidence and survival (Pritchard-Jones, 2006; Ward et al., 2014). However, the 0-14 year age range was also used to define pediatrics in some countries and international health organizations (Force et al., 2019). The tumour types were grouped according to the third edition of the International Childhood Cancer Classification, ICCC-3 (Steliarova-Foucher et al., 2005). The sixteen years study period was divided into three periods (2002-2006; 2007-2011; 2012-2017) to compare survival over time. 

The analyses were performed using R statistical software. Descriptive analyses, which include number of cases and its percentage, were conducted to evaluate the distribution of each variable. Survival time was defined as the period of time from diagnosis to death or end of follow-up. Patients who were still alive or lost to follow-up at the end of the study period were right-censored. Overall survival rates, defined as the period of time from diagnosis to death or end of follow-up due to any cause, were calculated using the Kaplan-Meier method. Univariate analysis, using the log-rank test, was used to examine the differences in survival between groups. Multivariate analysis, using the Cox Proportional Hazard (PH) regression model, was used to estimate the hazard ratios (HR) of associated factors on survival and to select the significant predictors for childhood cancer patients’ survival. Reference categories were selected logically as the first category (Male and Malay) or as the modal category (15-19 year age group and period 2012-2017). We examined the PH assumption and measured the goodness of fit for model adequacy. For all analyses, the level of statistical significance was set at 5%. Confidence intervals (C.I.) of 95% were reported where appropriate.

## Results

A total of 263 cases of childhood cancer registered during January 2002 to December 2017 were included in the analysis. The highest number of cases was recorded in year 2015 (n=28 cases) while the lowest was in year 2002 (n=8 cases), with an average of 16 cases per year. Of these 263 children and adolescents, 20.2% (n=53) had died within 1 year, and 28.1% (n=74) within 5 years of diagnosis. The mean and median ages at diagnosis were 9.4 years (standard deviation=6.8) and 9.0 (0-19) years, respectively. 

There were more male (55.5%) than female (44.5%) as shown in Table 1. Leukemia was the most common cancer (32.7%) followed by malignant epithelial neoplasms (13.7%), lymphomas (12.5%), CNS tumours (11.4%) and gonadal and germ cell neoplasms (7.2%) for children and adolescents aged 0-19. Majority of the childhood cancer patients were aged between 15-19 years (34.6%) followed by 1-4 years (25.5%), 5-9 years (15.2%) and 10-14 years (15.2%), and 0 year old (9.5%). Similar to the population ethnic group distribution in the country, most of the cases were among Malay ethnicity (89.7%), followed by Others (5.3%) and Chinese (4.9%). Majority of the cases were in the recent period 2012-2017 (44.1%). 

**Figure 1:**
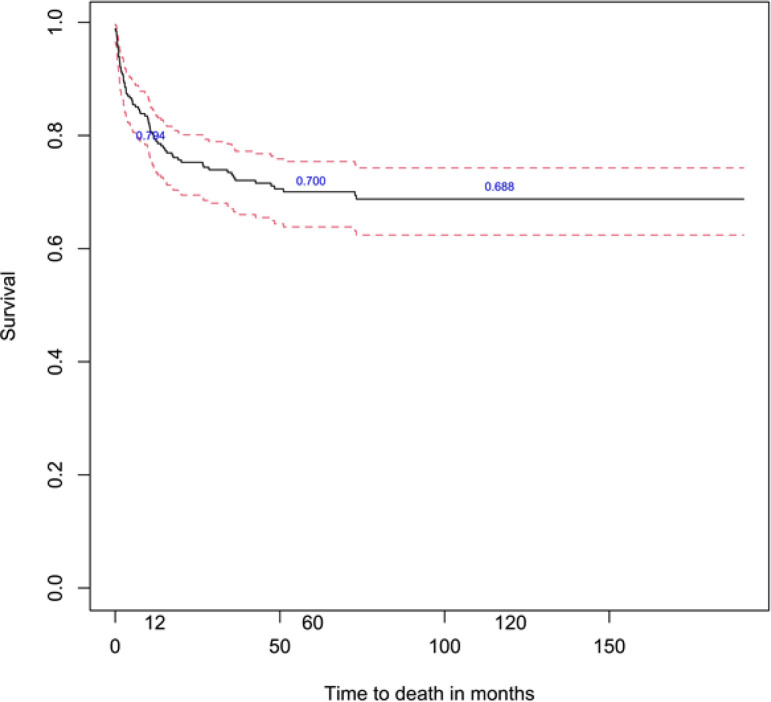
Overall Survival Estimates for Children and Adolescents Aged 0-19 Years Diagnosed with Childhood Cancer, with 95% Confidence Interval

**Figure 2:**
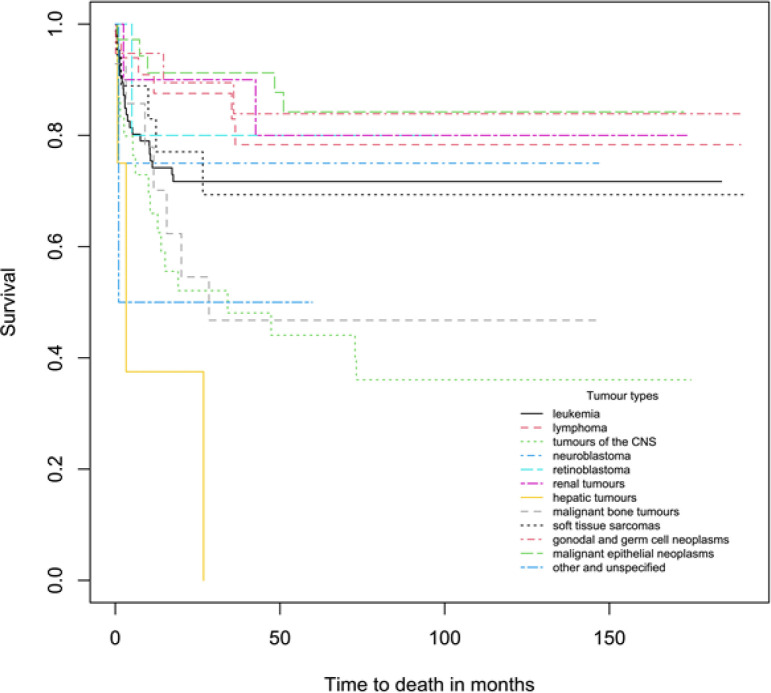
Survival Curves for Children and Adolescents Aged 0-19 Years by Tumour Types

**Figure 3:**
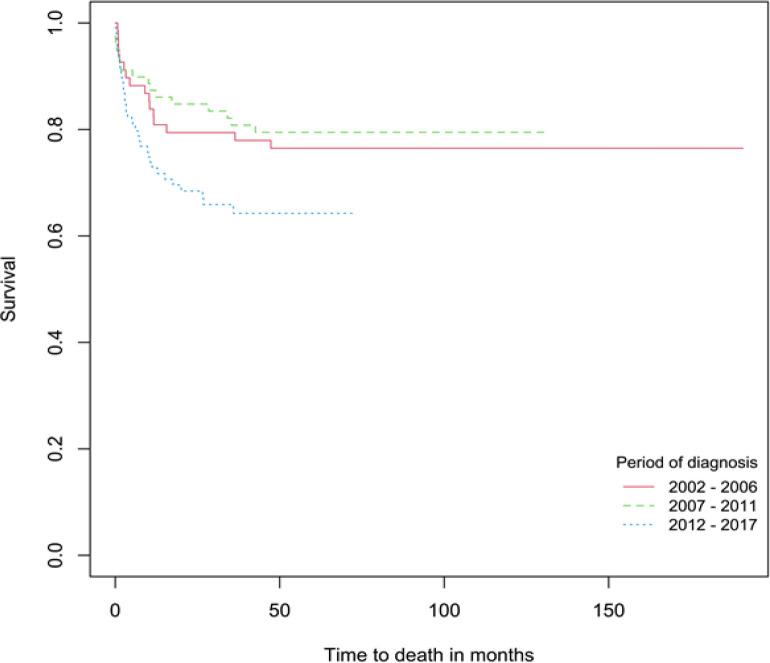
Survival Curves for Children and Adolescents Aged 0-19 Years by Period of Diagnosis

The 1-, 5- and 10-year overall survival rates of children and adolescents aged 0-19 years diagnosed with childhood cancers were 79.4% (95% C.I.: 73.9-83.8), 70.0% (95% C.I.: 63.8-75.4) and 68.8% (95% C.I.: 62.4-74.3) respectively (Figure 1). For children aged 0-14 years and adolescents aged 15-19 years, the 5-year overall survival rates were 74.4% and 61.6%, respectively (Table 1). Table 1 also presents the 5-year survival rates and p-values of the log-rank test for each covariate. This study found significant differences in childhood cancer patients’ survival between tumour types (p=0.0002) and period of diagnosis (p=0.0365). The overall 5-year survival was highest for malignant epithelial neoplasms (84.2%), followed by gonadal and germ cell neoplasms (83.9%), retinoblastoma (80.0%), renal tumours (80.0%), lymphomas (78.3%), neuroblastoma (75.0%), leukemias (71.7%), soft tissue sarcomas (69.3%), malignant bone tumours (46.8%) and CNS tumours (44.1%) (Figure 2; Table 1). Five-year survival for all childhood cancers combined improved from 76.5% (2002-2006) to 79.5% (2007-2011) but declined in 2012-2017 (64.2%) (Figure 3). We found no significant difference in survival between age groups, ethnicity and gender (p > 0.05). 

Table 2 reports the number of cases and 5-year survival rates for children and adolescent diagnosed with childhood cancers by age group and period of diagnosis. For children between 0-14 years of age, the three leading cancers were leukemia (n=71), CNS tumours (n=24) and lymphoma (n=17) while for adolescents aged 15-19 years, the three most common cancers were malignant epithelial neoplasms (n=28), lymphomas (n=16) and leukemias (n=15). 

Approximately 82.6% of those diagnosed with leukemia were children aged 0-14 with 5-year survival rate of 80.1%. Most of the children diagnosed with leukemia were in the 1-4 age group (38.4%) but they also had the highest 5-year survival rate of 90.9% compared to the other age groups with leukemia. The same trend could be seen for children diagnosed with CNS tumours where most were diagnosed in the 1-4 age group (30.0%) with the highest survival rate of 66.7%. Adolescents aged 15-19 years diagnosed with leukemia and CNS tumours had the lowest survival rate of 33.3% and 16.7% respectively, compared to the younger age groups of the same cancers. In comparison, children aged 0-14 diagnosed with CNS tumours had better survival rate (52.5%). Majority of patients diagnosed with lymphomas, malignant epithelial neoplasms, gonadal and germ cell neoplasms and soft tissue sarcomas were the adolescents aged 15-19 years. The highest 5-year survival rate for those diagnosed with lymphomas was in the 1-4 age group (100.0%) while for children diagnosed with malignant epithelial neoplasms, the highest 5-year survival rate was in the 10-14 age group (100.0%). 

**Figure 4:**
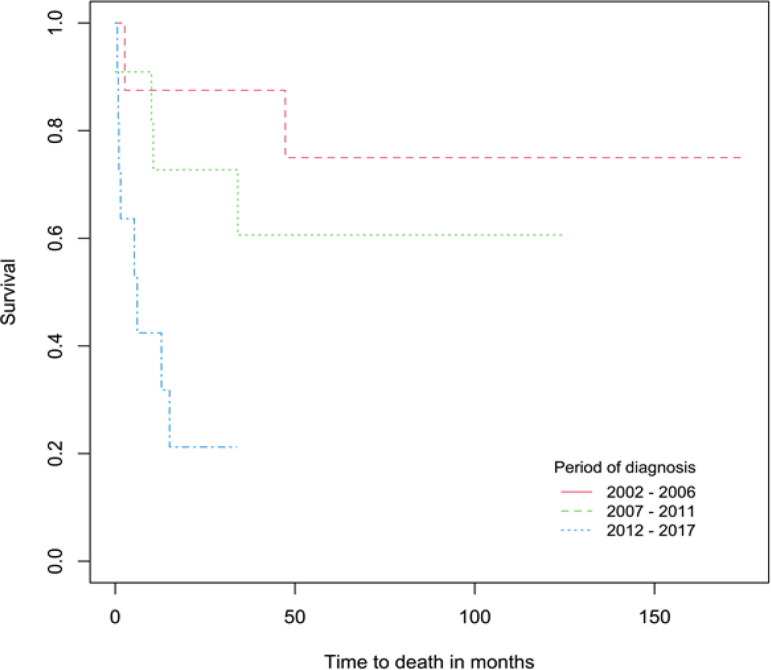
Survival Curves for Children and Adolescents Aged 0-19 Years Diagnosed with CNS Tumours by Period of Diagnosis

This study found majority of the cases were in the period 2012-2017 for all childhood cancers except for gonadal and germ cell neoplasms, neuroblastoma, renal tumours, malignant bone tumours and an equal number of cases for CNS tumours with period 2007-2011. For those diagnosed with leukemia, malignant epithelial neoplasms, and gonadal and germ cell neoplasms, the lowest 5-year survival rates was in period 2012-2017. For lymphomas, we found an improvement in survival from 50.0% in period 2002-2006 to 88.9% in 2007-2011 but declined to 85.0% in 2012-2017. For patients diagnosed with CNS tumours, the 5-year survival rate declined from 75.0% in 2002-2006 to 60.6% in 2007-2011. The 1-year survival rates for CNS tumours were lowest in 2012-2017 at 42.4% compared to periods 2002-2006 (87.5%) and 2007-2011 (72.7%), shown in figure 4. Children and adolescents diagnosed with neuroblastomas and malignant bone tumours showed higher survival in 2007-2011 compared to 2002-2006 but for those diagnosed with soft tissue sarcomas, the survival declined from 100.0% in 2002-2006 to 50.0% in 2007-2011 but improved to 57.1% in 2012-2017.

Cox PH regression analyses found that tumour types, age at diagnosis and period of diagnosis were significant factors for childhood cancer patients’ survival (p<0.05), shown in Table 3. No violations of PH assumptions were observed. For tumour types, as compared to a patient diagnosed with leukemia, the hazard ratios (HRs) for those diagnosed with lymphomas, gonodal and germ neoplasms, and malignant epithelial neoplasms were 0.34 (95% C.I.: 0.13-0.85), 0.16 (95% C.I.: 0.04-0.70) and 0.12 (95% C.I.: 0.03-0.41) respectively. Children diagnosed at aged 1-4 and 5-9 exhibited HRs of 0.24 (95% C.I.: 0.11-0.50) and 0.29 (95% C.I.: 0.12-0.66) respectively compared to adolescents. As for the period of diagnosis, compared to children and adolescents diagnosed in 2012-2017, HRs for those diagnosed in 2002-2006 and 2007-2011 were 0.51 (95% C.I.: 0.28-0.94) and 0.41 (95% C.I.: 0.22-0.76) respectively.

**Table 1:** Number of Cases, 5-Year Survival Rates and P-values of Log Rank Test by Age at Diagnosis, Ethnicity, Gender, Tumour Types and Period of Diagnosis of Childhood Cancers

	No. of cases	Percentage (%)	SR (95% C.I.)	Log rank test
Age at diagnosis				p=0.2780
0-14	172	65.4	74.4 (67.0-80.4)	
0	25	9.5	67.6 (45.4-82.3)	
1-4	67	25.5	81.1 (69.0-88.8)	
5-9	40	15.2	76.1 (58.9-86.8)	
10-14	40	15.2	66.7 (49.6-79.2)	
15-19	91	34.6	61.6 (50.1-71.3)	
Ethnicity				p=0.9170
Malay	236	89.7	70.1 (63.5-75.7)	
Chinese	13	4.9	76.9 (44.2-91.9)	
Others	14	5.3	63.5 (33.1-83.0)	
Gender				p=0.1910
Male	146	55.5	66.9 (58.2-74.2)	
Female	117	44.5	73.9 (64.6-81.1)	
Tumour types				p=0.0002*
Leukemias	86	32.7	71.7 (60.8-80.1)	
Lymphomas	33	12.5	78.3 (57.2-89.9)	
Tumours of the CNS	30	11.4	44.1 (25.5-61.2)	
Neuroblastoma	4	1.5	75.0 (12.8-96.1)	
Retinoblastoma	7	2.7	80.0 (20.4-96.9)	
Renal tumours	10	3.8	80.0 (40.9-94.6)	
Hepatic tumours	4	1.5	-	
Malignant bone tumours	14	5.3	46.8 (19.6-70.2)	
Soft tissue sarcomas	18	6.8	69.3 (40.6-86.2)	
Gonadal and germ cell neoplasms	19	7.2	83.9 (57.9-94.5)	
Malignant epithelial neoplasms	36	13.7	84.2 (65.9-93.2)	
Other and unspecified	2	0.8	-	
Period of diagnosis				p=0.0365*
2002-2006	68	25.9	76.5 (64.5-84.9)	
2007-2011	79	30	79.5 (68.7-86.9)	
2012-2017	116	44.1	64.2 (53.9-72.9)	

*statistically significant (p<0.05); SR, 5-year survival rate; C.I, confidence interval; CNS, Central Nervous System

**Table 2:** Number of Cases and 5-Year Overall Survival Rate for Children Diagnosed with Childhood Cancers by Age Group and Period of Diagnosis

Tumour types	Age at diagnosis												Period of diagnosis					
	0-14		0		1-4		5-9		10-14		15-19		2002-2006		2007-2011		2012-2017	
	n (%)	SR (%)	n (%)	SR (%)	n (%)	SR (%)	n (%)	SR (%)	n (%)	SR (%)	n (%)	SR (%)	n (%)	SR (%)	n (%)	SR (%)	n (%)	SR (%)
Leukemias	71 (82.6)	80.1	10 (11.6)	50	33 (38.4)	90.9	18 (20.9)	82.5	10 (11.6)	70	15 (17.4)	33.3	25 (29.1)	80	23 (26.7)	82.6	38 (44.2)	59.1
Lymphomas	17 (51.5)	73.2	1 (3.0)	-	6 (18.2)	100	3 (9.1)	66.7	7 (21.2)	57.1	16 (48.5)	80.8	4 (12.1)	50	9 (27.3)	88.9	20 (60.6)	85
Tumours of the CNS	24 (80.0)	52.5	4 (13.3)	50	9 (30.0)	66.7	7 (23.3)	51.4	4 (13.3)	25	6 (20.0)	16.7	8 (26.7)	75	11 (36.7)	60.6	11 (36.7)	-
Malignant epithelial neoplasms	8 (22.2)	100	0 (0.0)	-	0 (0.0)	-	0 (0.0)	-	8 (22.2)	100	28 (77.8)	79.7	9 (25.0)	100	10 (27.8)	100	17 (47.2)	80.1
Gonadal and germ cell neoplasms	8 (42.1)	87.5	3 (15.8)	100	1 (5.3)	-	0 (0.0)	-	4 (21.1)	100	11 (57.9)	80.8	5 (26.3)	100	8 (42.1)	87.5	6 (31.6)	80
Soft tissue sarcomas	7 (38.9)	85.7	0 (0.0)	-	2 (11.1)	100	5 (27.8)	80	0 (0.0)	-	11 (61.1)	53.7	4 (22.2)	100	6 (33.3)	50	8 (44.4)	57.1
Neuroblastoma	4 (100.0)	75	2 (50.0)	50	1 (25.0)	100	1 (25.0)	-	0 (0.0)	-	0 (0.0)	-	2 (50.0)	50	1 (25.0)	100	1 (25.0)	-
Retinoblastoma	7 (100.0)	80	2 (28.6)	100	5 (71.4)	75	0 (0.0)	-	0 (0.0)	-	0 (0.0)	-	0 (0.0)	-	1 (14.3)	100	6 (85.7)	75
Renal tumours	10 (100.0)	80	2 (20.0)	100	5 (50.0)	60	2 (20.0)	100	1 (10.0)	100	0 (0.0)	-	4 (40.0)	100	3 (30.0)	66.7	3 (30.0)	66.7
Hepatic tumours	3 (75.0)	-	0 (0.0)	-	2 (50.0)	-	1 (25.0)	-	0 (0.0)	-	1 (25.0)	-	1 (25.0)	-	0 (0.0)	-	3 (75.0)	-
Malignant bone tumours	12 (85.7)	50	1 (7.1)	100	2 (14.3)	50	3 (21.4)	100	6 (42.9)	16.7	2 (14.3)	-	5 (35.7)	20	7 (50.0)	71.4	2 (14.3)	-

SR, 5-year survival rate; CNS, Central Nervous System

**Table 3:** Hazard Ratios (HR) and 95% Confidence Intervals by Tumour Types, Age at Diagnosis and Period of Diagnosis

Variables	HR	95% C.I.	p-value
Tumour types			
Leukemias	1.00 (Reference)		
Lymphomas	0.34	0.13-0.85	0.0215*
Tumours of the CNS	1.9	0.97-3.72	0.0604
Neuroblastoma	1.25	0.16-9.51	0.8317
Retinoblastoma	0.71	0.09-5.43	0.7376
Renal tumours	0.74	0.17-3.23	0.6918
Hepatic tumours	2.42	0.69-8.51	0.1686
Malignant bone tumours	1.84	0.76-4.46	0.1776
Soft tissue sarcomas	0.61	0.22-1.68	0.3401
Gonadal and germ cell neoplasms	0.16	0.04-0.70	0.0152*
Malignant epithelial neoplasms	0.12	0.03-0.41	0.0008*
Other & unspecified	2.5	0.33-19.17	0.3774
Age at diagnosis			
0	0.61	0.26-1.45	0.2605
1-4	0.24	0.11-0.50	0.0002*
5-9	0.29	0.12-0.66	0.0031*
10-14	0.73	0.36-1.48	0.3791
15-19	1.00 (Reference)		
Period of diagnosis			
2002-2006	0.51	0.28-0.94	0.0318*
2007-2011	0.41	0.22-0.76	0.0046*
2012-2017	1.00 (Reference)		

HR, Hazard Ratio; C.I, Confidence Interval; *, statistically significant (p<0.05); p-value for Wald statistics; CNS, Central Nervous System

## Discussion

This is the first study in Brunei Darussalam looking at the survival rates and the associated factors for childhood cancer patients’ survival aged between 0-19 years of age. For all cancers combined, the overall survival rates of childhood cancer patients aged 0-19 years during 2002-2017 at 1-, 5- and 10-year were 79.4%, 70.0% and 68.8% respectively.

Although Brunei Darussalam has achieved the WHO’s target of 60% overall survival rate of childhood cancer, our overall 5-year survival for childhood cancer patients aged 0-19 years (70.0%) for 2002-2017 were found to be lower than that reported by published literatures from the United States at 83.0% (Ward et al., 2014) and Canada at 82.0% (Ellison et al., 2007) but comparable to Turkey at 67.8% (Kutluk and Yesilipek, 2018) and Singapore at 75% (Tan and Ha, n.d.). The 5-year survival rate for children aged 0-14 years for all cancers combined in this study was 74.4%, higher than developing countries in Asia such as Thailand (47.2%) (Bidwell et al., 2019) and China (71.9%) (Zheng et al., 2015), but lower than other developed countries such as South Korea (78.2%) (Park et al, 2016) and Australia (79.5%) (Baade et al., 2010). These disparities may be caused by multiple factors, with a country’s economic status being one contributor (Park, 2016). It is important to note that direct comparison with published survival rates internationally should be made with caution because of the different methodologies used, such as the age groups and time periods considered in the analyses.

Overall, among children and adolescents aged 0-19 years in Brunei Darussalam, the most common types of cancer were leukemias, malignant epithelial neoplasms, lymphomas and tumours of the CNS. However, the most common childhood cancers globally vary by age. Globally, cancers that were most common in children aged 0-14 years were leukemia, tumours of the CNS and lymphomas (Steliarova-Foucher et al., 2017), similar to our study. For adolescents aged 15-19 years, we found that the most common cancers were malignant epithelial neoplasms, followed by lymphomas and leukemia. Although globally, lymphomas were the most common cancer, followed by epithelial tumours and leukemia for adolescents aged 15-19 years, the most common cancer for adolescents in Oceania, east Asia, central America and the Caribbean and in white non-Hispanic children in the USA, was epithelial tumours and melanoma (Steliarova-Foucher et al., 2017), consistent with our study.

Leukemia contributed to roughly one-third of the childhood cancer cases in Brunei Darussalam, similar to that reported elsewhere (Bidwell et al., 2019; Park et al., 2016). Children aged 0-14 years diagnosed with leukemia had 5-year survival rate of 80.1% in this study. Japan reported 5-year survival rate of 83% (Nakata et al., 2018) while South Korea recorded 5-year survival rate of 75.4% (Park et al., 2016) for this age group. We found huge differences in survival among age groups for childhood leukemia, with highest survival for children aged 1-4 years while lowest survival for adolescents aged 15-19, similar to a study in the United States which reported the age group 15-19 continued to show the lowest survival for longer time, while the age group 1-4 showed a persistent highest survival (Holmes et al., 2012). Improvements in childhood leukemia survival over time have been reported internationally and have been suggested to be most likely due to advances in treatment, standardization of treatment protocols and improvements in diagnosis of leukemia (Bonaventure et al., 2017) 

Among the childhood cancers, for children and adolescents aged 0-19, the highest 5-year survival rate was observed in malignant epithelial neoplasms (84.2%) while the lowest survival was for CNS tumours (44.1%). Adolescents aged 15-19 years diagnosed with CNS tumours observed 5-year survival rate of 16.7%, much lower than studies reported internationally with estimates that range from 39.4% to 54.8% (Bidwell et al., 2019; Ballantine et al., 2017). The low survival rate observed in this study indicates a need for future follow-up studies focusing on the specific group of cancer, age groups and the associated risk factors. However, children aged 0-14 diagnosed with CNS tumours had survival rate of 52.5%, which is comparable to studies from Korea at 59.0% (Park et al., 2018), and Estonia at 49.5% (Paapsi et al., 2020) but lower than Australia at 71.0% (Baade et al., 2010). The survival estimates for CNS tumours may be somewhat affected by the use of different diagnostic methods and the classification of tumour behaviour (Peris-Bonet et al., 2006). When compared to children aged 0-14, adolescents observed higher survival rate when diagnosed with lymphomas but lower when diagnosed with malignant epithelial neoplasms. In general, these survival rates are higher than developing countries in Asia (Wiangnon et al., 2011, NCR, 2018) but they are still below those of the international progress (Desandes et al., 2014; Park et al. 2016). 

Majority of the childhood cancer cases were in the recent period (2012-2017). The increase in the number of childhood cancer cases could be due to the improvement in pediatric cancer registration, detection and reporting in that period. In 2013, Brunei Darussalam healthcare services adopted an electronic medical record system to enhance the accuracy, timeliness and completeness of the country’s cancer registry (Leong et al., 2019). The 5-year survival rate for all cancer sites combined improved slightly from 76.5% in period 2002-2006 to 79.5% in 2007-2011 but declined to 64.2% in the period 2012-2017. The lower overall survival rate for period 2012-2017 is contributed by the low survival rates for some cancers, especially leukemia and CNS tumours, as their 5- and 1-year survival rates were found to be 59.1% and 42.4%, respectively in that period. 

The following factors were significantly associated with lower hazard of death in this study: the presence of lymphomas, gonodal and germ cell neoplasms, and malignant epithelial neoplasms compared to leukemia; children aged 1-4 and 5-9 years compared to adolescents aged 15-19 years; and periods of diagnosis in 2002-2006 and 2007-2011 compared to 2012-2017. Similar to our study, Zouain-Figuerido et al (2013) reported higher age compared with the 1-4 age group was risk factor for lower survival rates in their study, in which their HR of mortality for adolescents aged 15-19 was 1.64 compared to children aged 1-4. 

This study has several limitations. Factors such as treatment, information on family history of cancer and cancer stage were associated with childhood cancer patients’ survival (Baade et al., 2010; Keramatinia et al., 2018; Kollerud et al., 2018). Information on these factors was not included in this study and should be explored in future studies. In addition, due to the rarity and small number of some cancers, five-year survival rate was not available from the statistical analysis. There was also presence of missing data, commonly seen in all retrospective studies. Despite these limitations, the findings of the present study provide important baseline measurements of childhood cancer survival in Brunei Darussalam. 

In conclusion, overall survival rates of childhood cancer and breakdown by types of cancers, age groups and time period found in this analysis provide baseline monitoring and evaluation of cancer control programmes, and the findings will be helpful for planning cancer control program strategies such as surveillance, screening, and treatment to improve childhood survival rates in Brunei Darussalam. Efforts to improve survival should be continued, especially for cancers with low survival rates such as CNS tumours. Effective health campaigns should be implemented to raise awareness on childhood cancers and health education programs emphasizing the importance of early diagnosis, infection control, and education of parents or primary caregivers. 
